# Long-term Potentiation at Temporoammonic Path-CA1 Synapses in Freely Moving Rats

**DOI:** 10.3389/fncir.2016.00002

**Published:** 2016-02-10

**Authors:** Jossina Gonzalez, Desiree M. Villarreal, Isaiah S. Morales, Brian E. Derrick

**Affiliations:** ^1^Department of Biology, University of Texas at San AntonioSan Antonio, TX, USA; ^2^UTSA Neurosciences Institute, University of Texas at San AntonioSan Antonio, TX, USA

**Keywords:** LTP, *in vivo*, current source density, CA1, temporoammonic path

## Abstract

Hippocampal area CA1 receives direct entorhinal layer III input via the temporoammonic path (TAP) and recent studies implicate TAP-CA1 synapses are important for some aspects of hippocampal memory function. Nonetheless, as few studies have examined TAP-CA1 synaptic plasticity *in vivo*, the induction and longevity of TAP-CA1 long-term potentiation (LTP) has not been fully characterized*.* We analyzed CA1 responses following stimulation of the medial aspect of the angular bundle and investigated LTP at medial temporoammonic path (mTAP)-CA1 synapses in freely moving rats. We demonstrate monosynaptic mTAP-CA1 responses can be isolated *in vivo* as evidenced by observations of independent current sinks in the *stratum lacunosum moleculare* of both areas CA1 and CA3 following angular bundle stimulation. Contrasting prior indications that TAP input rarely elicits CA1 discharge, we observed mTAP-CA1 responses that appeared to contain putative population spikes in 40% of our behaving animals. Theta burst high frequency stimulation of mTAP afferents resulted in an input specific and N-methyl-D-aspartate (NMDA) receptor-dependent LTP of mTAP-CA1 responses in behaving animals. LTP of mTAP-CA1 responses decayed as a function of two exponential decay curves with time constants (τ) of 2.7 and 148 days to decay 63.2% of maximal LTP. In contrast, mTAP-CA1 population spike potentiation longevity demonstrated a τ of 9.6 days. To our knowledge, these studies provide the first description of mTAP-CA1 LTP longevity *in vivo*. These data indicate TAP input to area CA1 is a physiologically relevant afferent system that displays robust synaptic plasticity.

## Introduction

Hippocampal memory function is thought to involve the relay of entorhinal layer II perforant path (PP) input to the dentate gyrus (DG) and the subsequent activation of the hippocampal “tri-synaptic loop” (Andersen et al., 1966, 1971a). Nevertheless, the *stratum lacunosum moleculare* of area CA1 pyramidal cells receive direct cortical input via the temporoammonic path (TAP; Ramon y Cajal, 1911; Lorente de Nó, 1934; Blackstad, 1958; Hjorth-Simonsen, 1972) which primarily arises from layer III pyramidal cells of the entorhinal cortex (Steward and Scoville, 1976).

Recent studies suggest TAP-CA1 input facilitates memory consolidation (Remondes and Schuman, 2004), place-specific firing of CA1 place cells (McNaughton et al., 1989; Mizumori et al., 1989; Brun et al., 2002, 2008; Nakashiba et al., 2008), spatial learning (Nakashiba et al., 2008), spatial recognition memory (Brun et al., 2002), temporal association memory (Suh et al., 2011), spatial working memory (Vago and Kesner, 2008), and intermediate-term spatial memory (Vago et al., 2007). TAP input is thought to modulate the induction of CA3-CA1 synaptic plasticity (Levy et al., 1998; Buzsáki, 2002; Remondes and Schuman, 2002; Judge and Hasselmo, 2004; Dudman et al., 2007; Xu et al., 2012) and regulate CA1 output (Empson and Heinemann, 1995a,b; Dvorak-Carbone and Schuman, 1999a; Remondes and Schuman, 2002). Furthermore, “match-mismatch” novelty detection and sequence prediction by CA1 pyramidal cells may result from comparisons of TAP inputs conveying current experience with previously stored associations relayed through Schaffer collaterals (Levy, 1989; Eichenbaum and Buckingham, 1990; Hasselmo and Schnell, 1994; Hasselmo and Wyble, 1997; Lisman, 1999; Lisman and Otmakhova, 2001; Hasselmo, 2005; Kumaran and Maguire, 2007; Vago and Kesner, 2008).

Long-term potentiation (LTP) encompasses a long lasting increase in synaptic efficacy following high frequency stimulation (Bliss and Gardner-Medwin, 1973; Bliss and Lømo, 1973). As LTP exhibits longevity and input specificity, this form of synaptic plasticity may represent a mechanism for memory storage and learning (Bliss and Gardner-Medwin, 1973; Bliss and Lømo, 1973; Teyler and Discenna, 1984; Bliss and Collingridge, 1993; Martinez and Derrick, 1996). Despite increasing evidence TAP-CA1 synapses are involved in some forms of memory, previous observations of TAP-CA1 LTP largely reflect *in vitro* investigations (Doller and Weight, 1985; Colbert and Levy, 1993; Dvorak-Carbone and Schuman, 1999b; Remondes and Schuman, 2002, 2003) that were unable to characterize LTP induction and longevity at specific medial or lateral TAP-CA1 synapses.

Given the paucity of data evaluating TAP-CA1 LTP *in vivo*, our study had two objectives: First, medial TAP-CA1 (mTAP-CA1) responses were characterized to determine if mTAP-CA1 responses can be isolated *in vivo*. Second, mTAP-CA1 LTP input specificity and longevity was investigated in freely moving animals. We demonstrate monosynaptic mTAP-CA1 responses can be selectively investigated *in vivo* without contamination of nearby current sources in the dentate gyrus (DG) and CA3. Our data also show mTAP-CA1 LTP is input specific, N-methyl-D-aspartate (NMDA) receptor-dependent, and persists approximately 2 weeks in behaving animals. As some animals exhibited TAP-CA1 responses that appeared to contain putative population spikes, our data also suggest TAP-CA1 synapses can contribute to pyramidal cell firing.

## Materials and Methods

### Subjects

The following experiments utilized 43 adult male Sprague Dawley rats (435–575 g, 4–5 months-old, Charles River Laboratories, Wilmington, MA, USA) individually housed at the University of Texas at San Antonio animal facility under a 12 h light/dark cycle with free access to water and a reduced feed diet. All experimental procedures adhered to the National Institutes of Health Animal Care and Use Guidelines and were approved by the Institutional Animal Care and Use Committee at the University of Texas at San Antonio.

### Chronic Electrode Implantation

**Figure 1:**
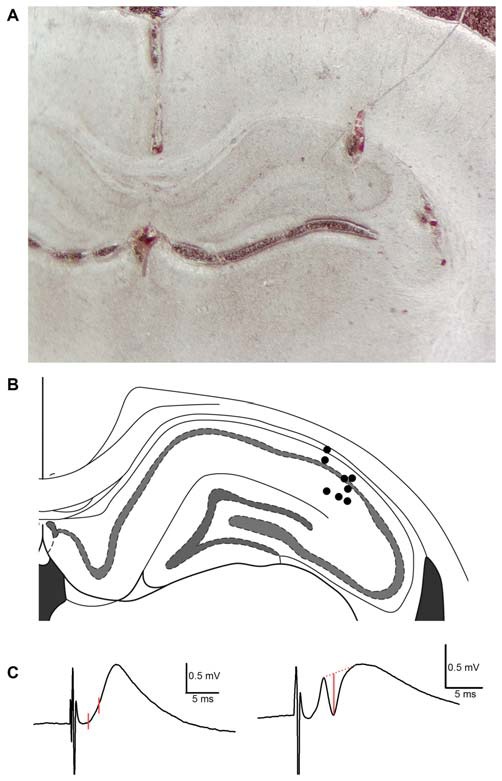
**Histological analysis of recording electrode placement in the proximal subfield of hippocampal area CA1 in subjects employed in TAP-CA1 LTP input specificity experiments. (A)** A representative example of histology obtained from a subject with a recording electrode in the proximal aspect of area CA1 and stimulating electrodes in the medial aspect of the angular bundle and contralateral CA3 region. **(B)** Electrode termination sites of eight out of nine animals are shown here as circles. Blood tracks and cell displacement enabled site determination of electrode termination. Histology was unavailable for one subject demonstrating electrophysiological criteria of monosynaptic mTAP-CA1 responses and included in TAP-CA1 LTP input specificity experiments. Figure adapted from Paxinos and Watson (2008). **(C)** mTAP-CA1 fEPSP slope and putative population spike measures are shown in red. fEPSP slopes were derived from the change in response amplitude over ~1 ms interval following the response onset. Amplitudes of putative population spikes were measured as the distance between the trough of the spike and a tangent line interleaving the peaks of the response. Calibration: 0.5 mV, 5 ms.

Rats received pre-operative doses of atropine (0.1 mg/kg, Sigma Aldrich, St. Louis, MO, USA) via an intraperitoneal (i.p.) injection and intramuscular administration of Baytril (3 mg/kg, UTSA Veterinary Services). Rats were deeply anesthetized with pentobarbital sodium (65 mg/kg, i.p., Sigma Aldrich, St. Louis, MO, USA) prior to undergoing chronic implantation of recording and stimulating electrodes under stereotaxic guidance. Supplemental i.p. injections of pentobarbital were administered, as needed, to sustain surgical levels of anesthesia. Body temperature was maintained at 37°C via a feedback regulated thermal heating pad and a rectal probe. As shown in Figure 1, a Teflon-coated, stainless steel recording electrode (0.005 inch in diameter and 1 MΩ impedance, A-M Systems) was implanted either in the *stratum pyramidale/stratum radiatum* or the *stratum lacunosum moleculare* regions of the proximal CA1 field (A/P −4.0 mm, M/L +3.0 mm, D/V −2.2 mm; Paxinos and Watson, 1994), respectively allowing collection of positive-going or negative-going CA1 field excitatory postsynaptic potentials (EPSPs) evoked by medial temporoammonic path (mTAP) stimulation (Steward, 1976; Wyss, 1981; Witter et al., 1989; Lopes da Silva et al., 1990). A twisted bipolar stainless steel stimulating electrode (0.005 inch in diameter and 1 MΩ impedance, A-M Systems) was situated in the medial aspect of the angular bundle (A/P +7.9 mm, M/L +4.2 mm, D/V −2.2 mm; Paxinos and Watson, 1994) to activate afferents from the medial entorhinal cortex (McNaughton and Barnes, 1977). Stimulation of the angular bundle activates both entorhinal layer II perforant path projections and entorhinal layer III TAP fibers (Witter et al., 1989; Leung et al., 1995; Canning and Leung, 1997). A second stimulating electrode was implanted within the contralateral CA3 region in order to activate commissural CA3 projections to the contralateral CA1 region (A/P +4.0 mm, M/L −3.0 mm, D/V −3.2 mm; Paxinos and Watson, 1994). Stimulation was delivered using a Grass S48 stimulator and a Grass PSIU6 constant current stimulus isolation unit. Evocation of mTAP-CA1 responses was verified by stereotaxic coordinates, audio localization of the CA1 pyramidal cell layer discharge and the occurrence of paired pulse facilitation (Speed and Dobrunz, 2009; Ito and Schuman, 2012; Aksoy-Aksel and Manahan-Vaughan, 2013), which contrasts the paired pulse depression observed at entorhinal layer II medial perforant path inputs to the DG (McNaughton, 1980) and area CA3 (Do et al., 2002).

In separate animals, medial perforant path-dentate gyrus (MPP-DG) responses, rather than commissural CA3-CA1 (cCA3-CA1) responses, were collected simultaneously by placing a recording electrode in the hilar region of the DG (A/P −3.5 mm, M/L −2.2 mm, D/V −3.0 mm; Paxinos and Watson, 1994). Evocation of MPP-DG responses was determined via stereotaxic coordinates, audio localization of the granule cell layer and observations of paired pulse depression (McNaughton, 1980).

Permanent indwelling electrodes were affixed with gold amphenol pins and stabilized in a head stage with stainless steel screws and dental acrylic. Animals were given Rimadyl (4 g, BID, BioServ) for 3 days post-operatively. Data collection commenced after a 2 week recovery period.

Responses were amplified with a P5 series Grass A/C amplifier, filtered at 0.3 Hz–3 kHz, digitized at 20 kHz and stored for off-line analysis (DataWave Technologies, Loveland, CO, USA). Following data collection, rats were sacrificed with Beuthanasia (350 mg/kg pentobarbital sodium, Henry Shein) prior to decapitation. Extracted brains were fixed in paraformaldehyde and sectioned for histological analysis of electrode positions (Figure 1). Animals were excluded if the recording electrode was not in area CA1 or if histological assessments were inconclusive.

### Experiments in Behaving Rats

All experiments were carried out in a sound-attenuating isolation chamber fitted with a video camera that permitted observations of the animal’s behavior.

Prior to data collection, current intensities evoking a half maximal mTAP-CA1 and cCA3-CA1 field EPSP were determined via input/output assessments of mTAP-CA1 and cCA3-CA1 slopes. TAP stimulation intensities ranged 32–458 μA whereas commissural CA3 input stimulation intensities ranged 39–480 μA. CA1 field EPSPs were evoked with constant biphasic current pulses (0.1 ms duration/phase) via an A365 stimulus isolator (World Precision Instruments, Sarasota, FL, USA). Slope measurements, reflecting the field EPSP rate of rise, were derived from the change in response amplitude over a 0.55–1.35 ms period following the response onset (Figure 1C). For animals that exhibited putative population spikes, field EPSP measurements were set at or prior to the initial peak of the population spike or, when possible, prior to any slope deviation that distinguished the initial field EPSP from an ensuing population spike.

Animals with chronic electrodes were initially given paired pulse stimulation of the medial TAP or commissural CA3 inputs at paired pulse intervals of 25, 50, 100 and 200 ms. For each paired pulse interval, five paired responses were collected every 15 s for subsequent slope analysis. Paired pulse ratios were derived from the slope of the second field EPSP divided by the slope of the first field EPSP. Paired pulse facilitation was defined as an average paired pulse ratio greater than one, whereas paired pulse depression comprised an average paired pulse ratio less than one. Given how mTAP-CA1 responses exhibit paired pulse facilitation (Ito and Schuman, 2012) and MPP-CA3 responses display paired pulse depression (Do et al., 2002), animals not demonstrating paired pulse facilitation of mTAP-CA1 field EPSPs were excluded from the study.

In addition, interpathway paired pulse stimulation was collected in freely moving animals to evaluate possible contamination of mTAP-CA1 inputs and cCA3-CA1 inputs by the alternate pathway. In these experiments, five baseline cCA3-CA1 responses were collected prior to evoking five CA1 responses where medial TAP stimulation preceded commissural CA3 input by 80 ms. Similarly, five baseline mTAP-CA1 responses were collected prior to recording five CA1 responses where commissural CA3 input stimulation preceded medial TAP input by 80 ms. To determine if heterosynaptic paired pulses evoke alterations in CA1 responses, the slope of the averaged interpathway paired pulse response was normalized with the slope of the averaged baseline response.

Daily mTAP-CA1 and cCA3-CA1 slope measures, comprising the average of ten mTAP-CA1 and cCA3-CA1 evoked responses, were collected for at least 5 days to obtain a stable daily baseline. Animals were excluded from subsequent LTP experiments if the mTAP-CA1 response was smaller than 0.35 mV, or if daily slope data deviated more than 15% of the overall baseline mean. To assess LTP input specificity, CA1 responses were evoked with 0.066 Hz alternating stimulation of the medial TAP and commissural CA3 fibers, both prior to and following theta burst stimulation (TBS). Following a stable 20 min baseline, three TBS epochs were applied to the angular bundle of awake animals at 5 min intervals where each TBS epoch was comprised of five theta burst trains of five 25 ms, 400 Hz bursts delivered every 200 ms with an inter-train interval of 15 s. mTAP-CA1 and cCA3-CA1 responses were collected for an additional hour after TBS. Both TBS delivery and evocation of mTAP-CA1 and cCA3-CA1 test responses employed half maximal intensities, as listed above.

LTP longevity assessments involved daily mTAP-CA1 and cCA3-CA1 response collection. Post-LTP dailies were recorded for a minimum of 7–10 days or until mTAP-CA1 field EPSPs decayed and remained at baseline for 3 consecutive days. In cases when LTP lasted more than 21 days, mTAP-CA1 and cCA3-CA1 responses were collected every third day until mTAP-CA1 slope measures returned to baseline for two consecutive recording sessions.

Following collection of LTP data, CA1 responses were recorded during a four pulse, 100 Hz burst stimulation of the angular bundle to characterize mTAP-CA1 responses as monosynaptic if field EPSPs followed high frequency pulses in a one-to-one manner (Lømo, 1971; McNaughton and Barnes, 1977; Yeckel and Berger, 1990). Data were confined to monosynaptic responses that displayed an onset latency ≤3 ms (Yeckel and Berger, 1990, 1995). Animals were excluded if mTAP-CA1 field EPSPs were not monosynaptic based on the above criteria.

To assess whether mTAP-CA1 LTP induction requires NMDA receptor activation, animals with DG and CA1 recording electrodes were given NMDA receptor antagonist (+)-5-methyl-10,11-dihydro-5H-dibenzo[a,d]cycloheptene-5,10-imine hydrogen maleate (MK-801; 2 mg/kg, Sigma-Aldrich, St. Louis, MO, USA) or water vehicle i.p. 1 h prior to TBS of the angular bundle in awake, freely moving animals. mTAP-CA1 and MPP-DG responses were simultaneously collected for an additional hour after TBS. Some MK-801 treated animals were used in subsequent water vehicle experiments 1 week after MK-801 data collection.

### Current Source Density (CSD) Experiments

Rats (*n* = 3) were anesthetized with urethane (1.5 g/kg, i.p.) prior to implanting a recording electrode in the DG to optimize the stimulating electrode position in the medial aspect of the angular bundle (A/P −8.1 mm, M/L +4.1 mm, D/V −2.5 mm; Paxinos and Watson, 1994). The recording electrode was subsequently positioned in CA3 *stratum oriens*. Current source density (CSD) analysis occurred in a bottom-to-top fashion (Leung et al., 1995) by collecting locally generated field EPSPs as the recording electrode was raised, in stepwise 50 μm intervals, across the lamina of hippocampal areas CA1 and CA3. For each depth interval, ten responses were evoked by 0.2 Hz stimulation of the angular bundle, digitized at 10 kHz and averaged to produce a representative field EPSP for subsequent one dimensional CSD analysis via the following second order differential equation (Yeckel and Berger, 1998):

$${\text{I}}_{\text{x}}\;=\;-\sigma (E_{\text{x}-\text{h}}-2E_{\text{x}}+E_{\text{x+h}})/4{\text{h}}^{2}$$

where I_x_ is the current source density at depth x, h is the depth interval (50 μm), *E*_x_ is the potential at depth x and σ is conductivity. σ was considered constant and CSD analyses were expressed in arbitrary units (V/mm^2^). CSD data were analyzed and plotted in SigmaPlot 12 (Systat Software, San Jose, CA, USA).

### Statistical Analysis

Slopes collected during TBS experiments were first normalized with respect to the average 15 min baseline slope and subsequently plotted in averaged 1 min intervals. LTP measurements were derived from field EPSP ratios of the normalized slope average obtained 56–60 min following TBS divided by the normalized slope average collected 5 min prior to TBS. Amplitudes of putative population spikes were measured as the distance between the trough of the spike and a tangent line interleaving the peaks of the response (Figure 1C). Statistical assessments of homosynaptic paired pulse facilitation and LTP input specificity data involved separate repeated measures two way analysis of variance (ANOVA). *Post hoc* Student-Newman-Keuls test indicated statistical significance at *p* < 0.05. mTAP-CA1 LTP daily data were averaged across animals for 30 days post LTP induction as mTAP-CA1 slope measures generally remained stable (i.e., 100 percent of baseline) after re-establishing baseline values for 3 consecutive days. Averaged daily data were plotted with an exponential curve fit (SigmaPlot 12, Systat Software, San Jose, CA, USA) to determine the LTP decay time constant (τ), defined as the time of decaying 63.2% of maximal LTP. Data values are expressed as mean ± SEM. A total of 22 out of 43 animals were excluded based on histology or failure to meet pre-defined criteria of monosynaptic mTAP-CA1 responses that were larger than 0.35 mV and exhibited paired pulse facilitation.

## Results

### Medial Temporoammonic Path-CA1 Responses can be Isolated *In Vivo* without Contamination from Other Inputs

**Figure 2:**
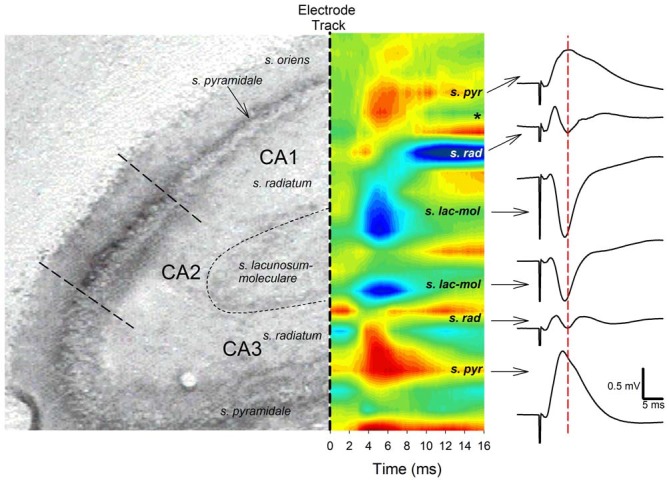
**Angular bundle stimulation evokes independent sinks (inward current) in CA1 and CA3 distal dendrites.** *Left panel*, A histological section of hippocampal areas CA1 and CA3 corresponding to the sites of sinks and sources (outward current) evoked by angular bundle stimulation in a urethane anesthetized rat (from Paxinos and Watson, 1994). *Middle panel*, Current source density (CSD) analysis for areas CA1 and CA3. Blue areas represent current sinks whereas red areas depict sources. Note distinct current sinks in proximal area CA1 and CA3 *stratum lacunosum moleculare* layers corresponding to medial TAP and MPP targets. Also note how CA3 sinks precede those of area CA1 by ~0.5–1 ms, suggesting distinct cortical afferents relay CA3 and CA1 synaptic responses. The *denotes the occurrence of polysynaptic activity, mostly likely originating from disynaptic MPP-CA3 and CA3-CA1 (Schaffer collateral) responses. *Right panel*, CA1 and CA3 responses phase reverse and exhibit different peak latencies. Calibration: 0.5 mV, 5 ms.

Both negative-going and positive-going TAP-CA1 field EPSPs were respectively examined via local field recordings in dendritic and somatic regions of an intact hippocampal formation, thus allowing evaluations of responses evoked near the site of synaptic activation and TAP-CA1 putative population spikes, respectively. We first verified the local generation of CA1 field EPSPs evoked by stimulation of the medial entorhinal cortex projections that course through the medial aspect of the angular bundle (McNaughton and Barnes, 1977). These examinations were crucial as distal apical dendrites of proximal CA1 and area CA3 are closely apposed (Ramon y Cajal, 1911; Lorente de Nó, 1934) and receive direct cortical inputs that produce otherwise indistinguishable negative-going responses (Colbert and Levy, 1992, 1993; Empson and Heinemann, 1995a,b; Wu and Leung, 1998; McMahon and Barrionuevo, 2002). Given how synaptic activation results in current flow that loops through the dendrite before exiting the soma (Llinás and Nicholson, 1974), negative-going field EPSPs recorded at the site of synaptic activation phase reverse at the soma. This shift in the field EPSP polarity enables the localization of “sinks” (inward current) and “sources” (outward current) through CSD analysis and serves to identify currents that underlie locally generated field EPSPs (Leung et al., 1995). As shown in Figure 2, both CA1 and CA3 field EPSPs phase reverse, suggesting angular bundle stimulation evokes locally generated responses in areas CA1 and CA3. In addition, CSD analysis demonstrates angular bundle stimulation results in separate sink-source pairs in areas CA1 and CA3 (Figure 2). Interestingly, CA3 sinks and field EPSP peak latency evoked by perforant path afferents occurred approximately 0.5–1 ms earlier than CA1 sinks and field EPSP peak latency produced by TAP afferents, suggesting distal dendritic CA3 and CA1 sinks are locally generated via independent afferent fibers originating from medial entorhinal cortex layer II stellate cells or layer III pyramidal cells (Steward and Scoville, 1976). Analysis of ten perforant path-CA3 evoked responses recorded from *stratum pyramidale/oriens* indicated an onset latency of 1.67 ± 0.05 ms (mean ± SEM) and a peak latency of 5.54 ± 0.04 ms, whereas mTAP-CA1 field EPSPs displayed an onset latency of 1.70 ± 0.04 ms and a peak latency of 6.45 ± 0.13 ms in a urethane anesthetized rat. mTAP-CA1 field EPSP peak latencies were significantly longer than perforant path-CA3 field EPSP peak latencies [*p* < 0.001, Kruskal-Wallis one way ANOVA on Ranks], whereas the onset latencies of both responses were comparable [*F*_(1,18)_ = 0.27, *p* > 0.05, one way ANOVA]. The differences in perforant path-CA3 and mTAP-CA1 sink and field EPSP peak latencies may suggest layer III medial TAP fibers demonstrate a slower conduction velocity than layer II medial perforant path fibers, as reported previously (Leung et al., 1995; Canning and Leung, 1997).

**Figure 3:**
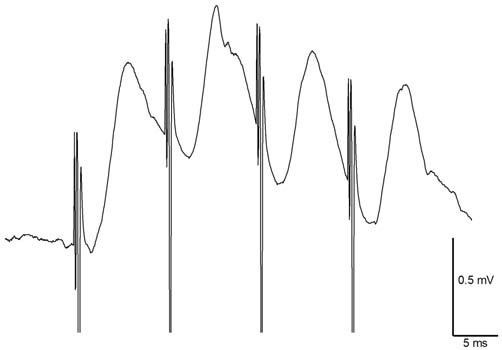
**Medial temporoammonic path (mTAP)-CA1 responses follow 100 Hz stimulation in freely moving rats suggesting mTAP-CA1 responses are monosynaptic.** Calibration: 0.5 mV, 5 ms.

In freely moving animals (*n* = 14), angular bundle stimulation evoked CA1 responses that measured 0.84 ± 0.1 mV in amplitude. mTAP-CA1 field EPSPs exhibited an average onset latency of 2.06 ± 0.1 ms and a peak latency of 5.34 ± 0.4 ms, suggesting mTAP-CA1 responses are monosynaptic. mTAP-CA1 field EPSP onset latency ranged 1.35–2.85 ms and peak latency ranged 4.25–7.85 ms. In addition, CA1 responses followed 100 Hz high frequency stimulation of the angular bundle (Figure 3), further indicating mTAP-CA1 responses are monosynaptically generated (Lømo, 1971; McNaughton and Barnes, 1977; Yeckel and Berger, 1990).

Although previous studies indicated direct cortical inputs fail to elicit CA1 population spikes (Andersen and Loyning, 1962; Colbert and Levy, 1992; Empson and Heinemann, 1995a,b; Chevaleyre and Siegelbaum, 2010), 6 out of 14 freely moving animals exhibited mTAP-CA1 field EPSPs that appeared to contain putative population spikes which are thought to reflect the synchronous discharge of a cell population (Andersen et al., 1971b; Lømo, 1971). These TAP-induced putative population spikes displayed an average latency of 4.26 ± 0.2 ms.

**Figure 4:**
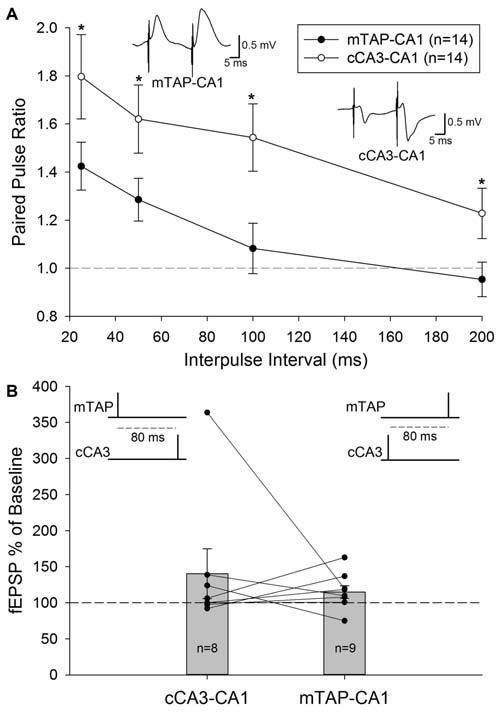
**Medial temporoammonic-CA1 responses can be isolated without contamination from other perforant path targets or CA1 afferents in freely moving animals. (A)** Paired pulse ratios were determined for both mTAP-CA1 (•, *n* = 14) and cCA3-CA1 (°, *n* = 14) responses collected at interpulse intervals of 25, 50, 100 and 200 ms. A *denotes *p* < 0.05 [repeated measures two way ANOVA]. Inset responses depict representative examples of mTAP-CA1 and cCA3-CA1 paired pulse facilitation following a 25 ms interpulse interval. Calibration: 0.5 mV, 5 ms. **(B)** Heterosynaptic facilitation is not reliably observed with heterosynaptic paired pulse stimulation of CA1 afferents in freely moving animals. Normalized cCA3-CA1 (*n* = 8) and mTAP-CA1 (*n* = 9) field EPSPs evoked with a heterosynaptic paired pulse stimulation interval of 80 ms. Data are represented as mean ± SEM. Symbols (•) reflect individual experiments and symmetrical data recordings of individual animals are shown as lines.

Unlike the paired pulse depression observed at entorhinal layer II medial perforant path inputs to the DG (McNaughton, 1980) and area CA3 (Do et al., 2002), paired pulse facilitation is observed in entorhinal layer III mTAP projections to area CA1 (Ito and Schuman, 2012; Aksoy-Aksel and Manahan-Vaughan, 2013). Our examinations of homosynaptic paired pulse stimulation of CA1 afferents demonstrate both mTAP-CA1 (*n* = 14) and cCA3-CA1 (*n* = 14) responses exhibit paired pulse facilitation in freely moving animals (Figure 4A). Both positive-going and negative-going mTAP-CA1 responses displayed paired pulse facilitation with 25–50 ms interpulse intervals. Accordingly, observations of paired pulse facilitation indicate mTAP-CA1 responses are not volume conducted from the DG or area CA3. Consistent with previous studies (Speed and Dobrunz, 2009), cCA3-CA1 responses displayed larger paired pulse ratios than mTAP-CA1 responses. A repeated measures two way ANOVA revealed significant differences in paired pulse ratios among the two pathways [*F*_(1,12)_ = 7.52, *p* < 0.05] and interpulse intervals [*F*_(3,36)_ = 24.97, *p* < 0.001]. In contrast, no significant interaction occurred between pathway and interpulse interval covariates [*F*_(3,35)_ = 0.19, *p* > 0.05, repeated measures two way ANOVA]. In addition to indicating medial TAP and CA3 commissural inputs are physiologically distinct, these findings suggest mTAP-CA1 responses can be recorded in intact animals without contamination from perforant path responses generated from other subfields of the hippocampal formation.

A subset of the 14 freely moving animals that demonstrated monosynaptic mTAP-CA1 and cCA3 responses underwent subsequent examination of heterosynaptic paired pulse stimulation of CA1 afferents. These evaluations did not produce reliable facilitation of cCA3-CA1 slopes when medial TAP input preceded cCA3-CA1 stimulation by 80 ms (*n* = 8; Figure 4B). Heterosynaptic facilitation was not observed when cCA3-CA1 input preceded medial TAP stimulation by 80 ms (*n* = 9; Figure 4B). These data suggest medial TAP and commissural CA3 inputs independently evoke CA1 responses. Together, these data suggest mTAP-CA1 field EPSPs can be isolated *in vivo* without contamination from other CA1 afferents or other perforant path targets.

### Medial Temporoammonic Path-CA1 Synapses Exhibit an Input Specific LTP in Behaving Animals

**Figure 5:**
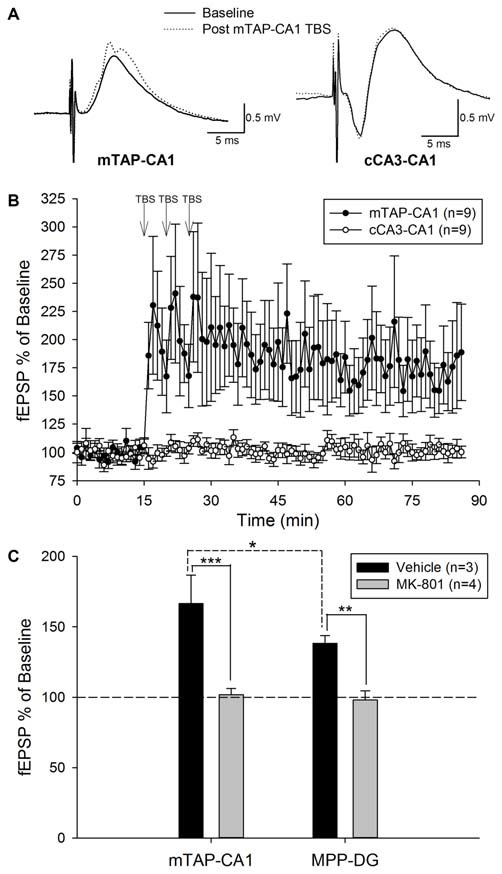
**Theta burst stimulation (TBS) of the medial temporoammonic path induces an input specific, NMDA receptor dependent LTP at mTAP-CA1 synapses in awake, freely moving rats. (A)** Representative examples of mTAP-CA1 and cCA3-CA1 responses collected during baseline (solid line) and 1 h after TBS (dashed line). Calibration: 0.5 mV, 5 ms. **(B)** mTAP-CA1 LTP is input specific as only mTAP-CA1 slopes (•), exhibited significant potentiation, whereas cCA3-CA1 slope (°) recordings from the same animals were unaffected after TBS (*n* = 9). **(C)** mTAP-CA1 and MPP-DG field EPSPs were collected in animals given either vehicle (*n* = 3) or MK-801 (2 mg/kg, *n* = 4) at least 1 h prior to TBS of the medial aspect of the angular bundle. Bar plots represent field EPSP ratios (mean ± SEM) derived from the average normalized slopes obtained 56–60 min post TBS divided by the average normalized slopes collected 5 min prior to TBS. Vehicle treated animals demonstrated mTAP-CA1 field EPSP ratios that were significantly larger than MPP-DG field EPSP ratios [*p < 0.05, *post hoc* Student-Newman-Keuls]. MK-801 significantly reduced field EPSP ratios [**p < 0.01, ***p < 0.001, two way ANOVA], suggesting NMDA receptor antagonism blocked LTP induction at mTAP-CA1 and MPP-DG synapses. These data indicate NMDA receptor activation is required for LTP induction at mTAP-CA1 and MPP-DG synapses.

Our preliminary studies of mTAP-CA1 response isolation *in vivo* allowed ensuing examinations of mTAP-CA1 synaptic plasticity in freely moving rats. Daily responses were recorded between 10:00 am and 12:00 pm to minimize circadian-related differences in field EPSPs (Cauller et al., 1985). Five of the fourteen animals with isolated mTAP-CA1 responses were excluded from subsequent LTP experiments due to a lack of baseline stability in daily recordings of mTAP-CA1 slopes. Nine animals exhibited a stable 5 day baseline of mTAP-CA1 slopes prior to TBS of the medial aspect of the angular bundle. These animals had accompanying cCA3-CA1 responses that enabled mTAP-CA1 LTP input specificity analysis. mTAP-CA1 slopes exhibited a robust potentiation measuring 170.9 ± 33 percent of baseline 1 h after TBS of the angular bundle in awake rats (Figures 5A,B). In the four animals with baseline mTAP-CA1 responses that appeared to contain putative population spikes, TBS potentiated population spike amplitudes measured 1 h after TBS (345 ± 139 percent of baseline, *F*_(1,3)_ = 24.01, *p* < 0.05, repeated measures one way ANOVA). Two out of four animals did not show concomitant potentiation of mTAP-CA1 fEPSPs. Observations of mTAP-CA1 population spike potentiation demonstrated decreases in mTAP-CA1 spike onset [*F*_(1,3)_ = 8.22, *p* = 0.06, repeated measures one way ANOVA] and population spike latency [*F*_(1,3)_ = 30.77, *p* < 0.05, repeated measures one way ANOVA] following LTP induction. Baseline mTAP-CA1 spike onset and population spike latency, on average, respectively measured 3.61 ± 0.38 ms and 5.0 ± 0.41 ms. Post TBS mTAP-CA1 spike onset and population spike latency measured 3.11 ± 0.20 ms and 4.21 ± 0.27 ms, respectively. Two additional animals demonstrated mTAP-CA1 putative population spikes following TBS, suggesting potentiated responses reached the threshold for medial TAP-mediated discharge of CA1 neurons. Contrasting the observed potentiation of mTAP-CA1 responses, cCA3-CA1 slopes measured 100.8 ± 3.9 percent of baseline 1 h after TBS of the angular bundle in freely moving animals (Figures 5A,B). A repeated measures two way ANOVA of normalized mTAP-CA1 and cCA3-CA1 slope averages obtained 5 min prior to TBS and 56–60 min following TBS indicate a significant pathway and baseline vs. post TBS slope interaction [*F*_(1,8)_ = 5.37, *p* < 0.05, *n* = 9]. These data suggest TBS of the TAP induces an input specific mTAP-CA1 LTP in awake, freely moving animals.

**Figure 6:**
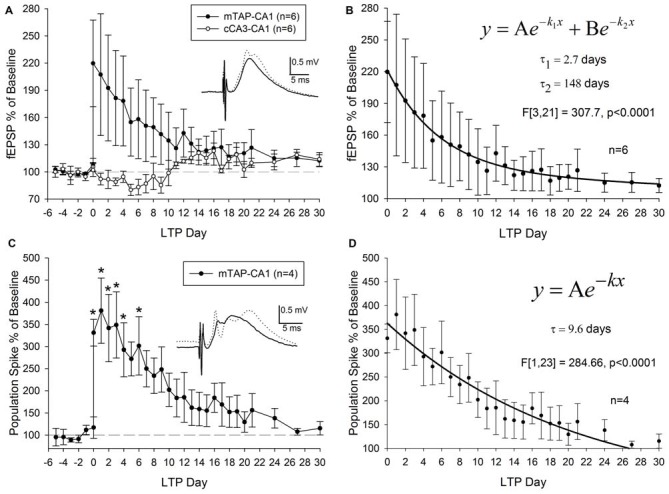
**mTAP-CA1 synapses and putative population spikes exhibit an enduring form of long-term potentiation (LTP) in freely moving rats. (A)** Daily mTAP-CA1 (•, *n* = 6) and cCA3-CA1 (°, *n* = 6) slope analysis. Inset: baseline (solid line) and post-TBS (dashed line) mTAP-CA1 responses. Calibration: 0.5 mV, 5 ms. Three out of six animals exhibited heterosynaptic long-term depression following TBS of the medial TAP. **(B)** Curve fitting of post LTP daily slope averages indicate mTAP-CA1 LTP decay (*n* = 6) is represented as a sum of two exponential curves with time constants (τ) of 2.7 days and 148 days to decay 63.2% of maximal LTP. **(C)** Daily mTAP-CA1 putative population spike amplitude analysis in animals that exhibited population spike potentiation following TBS (*n* = 4). Significant differences between post- and pre-tetanus putative population spike amplitudes persist for 5 days after LTP induction [*p < 0.05, repeated measures one way ANOVA]. Inset: baseline (solid line) and post-TBS (dashed line) mTAP-CA1 responses. Calibration: 0.5 mV, 5 ms. **(D)** Curve fitting of post LTP daily mTAP-CA1 putative population spike amplitude averages indicates population spike potentiation decays exponentially with a time constant (τ) of 9.6 days to decay 63.2% of maximal LTP.

Three out of nine animals did not demonstrate a potentiation of mTAP-CA1 slopes and were not included in subsequent analysis of LTP longevity. Thus, mTAP-CA1 LTP longevity was assessed in six animals that received daily response collection after LTP induction. This cohort consisted of four animals with positive-going and two animals with negative-going mTAP-CA1 field EPSPs, respectively reflecting recording electrode placement in the pyramidal cell layer/*stratum radiatum* or *stratum lacunosum moleculare* of area CA1. LTP of positive-going mTAP-CA1 field EPSPs measured 183.8 ± 60 percent of baseline and lasted 12 ± 8 days before decaying within a <15% deviation from baseline, a pre-defined criteria that we used to indicate a stable baseline. On average, LTP of negative-going mTAP-CA1 field EPSPs measured 235 percent of baseline and lasted 24.5 days before decaying within a <15% deviation from baseline. As no significant difference was observed for LTP measured 1 h after TBS [*F*_(1,4)_ = 0.23, *p* > 0.05, one way ANOVA, *n* = 6] or LTP longevity [*F*_(1,4)_ = 0.57, *p* > 0.05, one way ANOVA, *n* = 6] among positive-going and negative-going mTAP-CA1 field EPSPs, these data were combined for subsequent LTP decay analysis (Figures 6A,B).

On average, LTP of mTAP-CA1 field EPSPs endured 16 ± 8 days before decaying within a <15% deviation from baseline, a pre-defined criteria for a stable baseline. A significant positive correlation was observed between mTAP-CA1 LTP measured 1 h after TBS and LTP longevity (*r^2^* = 0.93, *p* < 0.05). Thus, large mTAP-CA1 LTP required more time to decay to baseline, similar to LTP decay tendencies observed at MPP-DG synapses (Barnes, 1979). Interestingly, curve fitting of mTAP-CA1 LTP daily averages indicated LTP decay consisted of a sum of two exponential decay curves [*F*_(3,21)_ = 307.7, *p* < 0.0001, ANOVA] with time constants (τ) of 2.7 and 148 days to decay 63.2% of maximal LTP (Figure 6B), suggesting mTAP-CA1 LTP decay is composed of fast and slow exponential decay components. We also examined LTP longevity of putative mTAP-CA1 population spikes via separate analyses of spike amplitudes in four animals that demonstrated stable putative population spikes over a 5 day baseline preceding TBS-induced observations of mTAP-CA1 spike potentiation (Figures 6C,D). mTAP-CA1 population spike LTP decay (*n* = 4) consisted of a single exponential decay component [*F*_(1,23)_ = 284.66, *p* < 0.0001, ANOVA] with a time constant of 9.6 days to decay 63.2% of maximal LTP (Figure 6D).

Although heterosynaptic long-term depression (LTD) at cCA3-CA1 synapses was not observed 1 h after mTAP-CA1 field EPSP LTP induction (Figures 5A,B), 50% of the animals with both mTAP-CA1 and cCA3-CA1 responses exhibited a slow developing heterosynaptic LTD in the days following TBS of the angular bundle (Figure 6A). Interestingly, next day observations of heterosynaptic LTD were correlated with small-to-moderate mTAP-CA1 LTP magnitudes measured 1 h after TBS (Spearman correlation analysis, *p* < 0.05, *n* = 6).

We next examined the receptor mechanisms involved in mTAP-CA1 LTP induction via simultaneous comparisons of the effects of NMDA receptor antagonist on medial perforant path-dentate gyrus (MPP-DG) and mTAP-CA1 LTP induction. As previous studies demonstrate MPP-DG LTP is blocked by NMDA receptor antagonists (Bramham et al., 1991), MPP-DG responses served as a positive control for assessing the NMDA receptor dependance of mTAP-CA1 LTP in freely moving rats. In naïve animals (*n* = 4), MPP-DG and mTAP-CA1 LTP induction was blocked by administration of MK-801 (2 mg/kg), a noncompetitive NMDA receptor antagonist, 1 h prior to TBS (Figure 5C). MK-801 treated animals (*n* = 4) demonstrated MPP-DG and mTAP-CA1 slopes respectively measuring 98.0 ± 6.5 and 101.8 ± 4.3 percent of baseline 1 h after TBS of the angular bundle. In contrast, vehicle treated animals (*n* = 3) demonstrated LTP of both MPP-DG and mTAP-CA1 slopes respectively measuring 138.3 ± 5.5 and 166.5 ± 20.2 percent of baseline 1 h after TBS of the angular bundle (Figure 5C). A two way ANOVA revealed a significant drug effect (vehicle vs. MK-801) on MPP-DG and mTAP-CA1 LTP magnitude [*F*_(1,10)_ = 40.93, *p* < 0.001], suggesting NMDA receptor antagonism blocked LTP induction at MPP-DG and mTAP-CA1 synapses. No significant drug and subfield (DG vs. CA1) interaction was demonstrated [*F*_(1,10)_ = 2.21, *p* > 0.05, two way ANOVA]. *Post hoc* Student-Newman-Keuls analysis demonstrated a significant difference in MPP-DG and mTAP-CA1 LTP magnitude of vehicle treated animals. While this observation suggests mTAP-CA1 synapses express a larger LTP than MPP-DG synapses, these differences in LTP magnitude may reflect a disparity in the current required to evoke a half-maximal responses at both MPP-DG and mTAP-CA1 synapses simultaneously. As these studies employed stimulation intensities that evoked a half-maximal mTAP-CA1 response, the range of MPP-DG LTP increases may be limited if evoked MPP-DG responses were not half-maximal prior to TBS. Nonetheless, these data suggest LTP induction is NMDA receptor dependent at both MPP-DG and mTAP-CA1 synapses *in vivo*. Taken together, these data demonstrate mTAP-CA1 LTP is input specific, requires NMDA receptor activation, and persists more than 2 weeks in behaving animals.

## Discussion

The main findings of these studies are that monosynaptic mTAP-CA1 responses can be isolated *in vivo* and exhibit input specific, NMDA receptor-dependent LTP that persists more than 2 weeks in behaving animals. As the validity of *in vivo* electrophysiological investigation is premised on isolating specific responses, one crucial objective of our studies was characterizing and confirming whether mTAP-CA1 responses can be isolated within intact animals. Several facets of our data suggest mTAP-CA1 responses can be selectively studied *in vivo*. First, mTAP-CA1 responses are locally generated as these field EPSPs exhibited both phase reversal and a short latency current sink-source pair in the *stratum lacunosum moleculare* and *stratum pyramidale* in area CA1. Second, both mTAP-CA1 responses and current sinks display longer peak latencies as compared to MPP-CA3 responses and current sinks, consistent with previous investigations indicating the TAP displays a slower conduction velocity than the perforant path afferents originating in layer II of the entorhinal cortex and projecting to the DG and area CA3 (Leung et al., 1995; Canning and Leung, 1997). Third, our observations of paired pulse facilitation at mTAP-CA1 synapses in freely moving animals indicate these responses are not volume conducted from the DG or area CA3 as previous reports indicate MPP afferents produce paired pulse depression in the DG (McNaughton, 1980) and area CA3 (Do et al., 2002). Fourth, heterosynaptic facilitation was not reliably observed among CA1 afferents and TAP TBS potentiated only those CA1 responses evoked by TAP inputs. These results suggest TAP and commissural CA3 inputs elicit independent responses that can be isolated in a freely moving animal. Although previous studies demonstrate heterosynaptic interactions between TAP- and CA3-CA1 EPSPs (Remondes and Schuman, 2002; Ang et al., 2005; Jarsky et al., 2005; Dudman et al., 2007; Takahashi and Magee, 2009), such interactions were not observed in our studies. However, it is possible that these interactions may not be readily apparent in local field potentials or extracellular population spikes. Fifth, mTAP-CA1 responses were able to follow high frequency stimulation. Taken together, these data indicate TAP-CA1 responses are monosynaptically generated, input specific and can be observed without contamination from other CA1 afferents or other perforant path targets *in vivo*.

The question of the ability of TAP inputs to elicit CA1 cell firing is important. CA1 pyramidal cells exhibit preserved place specific firing following lesions of the DG (McNaughton et al., 1989) or other manipulations that impair neurotransmission of CA3 inputs to area CA1 (Mizumori et al., 1989; Brun et al., 2002; Nakashiba et al., 2008). This suggests spatial information can be transmitted to the CA1 region by inputs other than those arising from CA3. Although the TAP afferents from the medial entorhinal cortex are the most likely source of place cell activity (Mizumori et al., 1989; Brun et al., 2002; Nakashiba et al., 2008), prior studies suggest TAP inputs are too weak to elicit CA1 discharge (Levy et al., 1995) given prior demonstrations of significant voltage attenuation of TAP-CA1 EPSPs (Golding et al., 2005) and the concomitant inhibition evoked by TAP inputs (Empson and Heinemann, 1995a,b). TAP stimulation has been shown to rarely evoke CA1 pyramidal cell discharge *in vitro* (Colbert and Levy, 1992; Empson and Heinemann, 1995a,b; Chevaleyre and Siegelbaum, 2010) or in urethane anesthetized animals (Andersen and Loyning, 1962; Gloor et al., 1962; Leung et al., 1995). In contrast, we observed mTAP-CA1 responses that displayed putative population spikes in freely moving animals, and these TAP-induced putative population spikes demonstrated common characteristics of population spike LTP such as reductions in spike latency and increases in spike amplitudes following TBS (Bliss and Gardner-Medwin, 1973; Bliss and Lømo, 1973). Our observations of possible population spikes in mTAP-CA1 field EPSPs support a previous demonstration of TAP-CA1 population spikes in guinea pigs (Doller and Weight, 1985) and are consistent with prior demonstrations that TAP inputs may sustain place field activity (McNaughton et al., 1989; Mizumori et al., 1989; Brun et al., 2002, 2008).

Supporting prior studies suggesting TAP-CA1 input is involved in certain forms of hippocampal memory function (Brun et al., 2002; Remondes and Schuman, 2004; Vago et al., 2007; Nakashiba et al., 2008; Vago and Kesner, 2008; Suh et al., 2011), recent studies have shown mTAP-CA1 synapses exhibit LTP that lasts more than 24 h following high frequency stimulation of the medial TAP in freely moving animals (Aksoy-Aksel and Manahan-Vaughan, 2013). Our data extend these findings by showing mTAP-CA1 LTP is input specific, NMDA receptor dependent and persists more than 2 weeks. Although we demonstrate *in vivo* mTAP-CA1 LTP induction requires NMDA receptor activation, previous *in vitro* studies suggest TAP-CA1 LTP displays only a partial dependance for NMDA receptors, where the concomitant NMDA receptor-independent potentiation requires activation of voltage-dependent calcium channels (Golding et al., 2002; Remondes and Schuman, 2003; Ahmed and Siegelbaum, 2009). What could underlie this difference in the requirement for NMDA receptor activation in TAP-CA1 LTP induction *in vivo* and *in vitro*? One likely reason is that it is difficult to stimulate selectively either medial or lateral TAP fibers in the slice preparation, as both sets of fibers course through the *stratum lacunosum moleculare* of area CA1, the site of stimulation employed in most *in vitro* studies. As lateral perforant path synapses in the DG (Bramham et al., 1991) and area CA3 (Do et al., 2002) demonstrate a NMDA receptor-independent LTP, it is possible that coactivation of both medial and lateral TAP inputs in these studies may have induced a NMDA receptor-independent LTP at lateral TAP-CA1 synapses (Golding et al., 2002; Remondes and Schuman, 2003; Ahmed and Siegelbaum, 2009). In this view, our observations showing a complete NMDA receptor dependance of mTAP-CA1 LTP may reflect a selective activation of medial TAP afferents *in vivo.* This would suggest that medial and lateral TAP synapses in area CA1 may exhibit LTP induction mechanisms identical to their medial and lateral perforant path counterparts in the DG and CA3. Future studies will be necessary to determine if this is indeed the case, with lateral TAP inputs displaying an LTP induction mechanism that does not require the activation of NMDA receptors (Bramham et al., 1991; Do et al., 2002).

LTP develops in three phases or forms (Abraham, 1990; Abraham and Otani, 1991; Raymond, 2007) known as LTP1, LTP2 and LTP3. Unlike the protein synthesis independent LTP1 that decays within hours of LTP induction, LTP2 and LTP3 represent longer lasting forms of potentiation that require protein synthesis of pre-existing or *de novo* messenger RNA (Krug et al., 1984; Otani et al., 1989; Abraham and Otani, 1991; Raymond, 2007), respectively. Contrasting the 6–8 day LTP longevity observed at PP-DG synapses (Barnes, 1979; Villarreal et al., 2002; Davis et al., 2004), our data suggests mTAP-CA1 LTP lasts, on average, more than 2 weeks after LTP induction. Similar to previous examinations of LTP decay at PP-DG synapses (Racine et al., 1983), we show mTAP-CA1 LTP decay is represented as a sum of two exponential decay curves consisting of a fast and slow exponential decay component. While the 2.7 day time constant of the fast component of mTAP-CA1 LTP decay is comparable to the 3.5 day LTP2 decay time constant at PP-DG synapses (Abraham and Otani, 1991), the 148 day time constant of the slow component of mTAP-CA1 LTP decay is strikingly different from the 20.3 day LTP3 decay time constant at PP-DG synapses (Abraham and Otani, 1991). Thus, mTAP-CA1 LTP longevity is greater than PP-DG LTP longevity.

We show TBS of mTAP-CA1 inputs also induced robust potentiation of putative population spikes in six animals, with two of these animals not exhibiting a putative population spike prior to LTP induction. Interestingly, mTAP-CA1 population spike potentiation decay properties contrasted that of field EPSP LTP decay, suggesting independent or additional mechanisms mediate the deterioration of potentiated population spikes. Unlike the LTP decay of mTAP-CA1 field EPSPs, potentiated mTAP-CA1 putative population spikes decayed with a single exponential decay component and a time constant of 9.6 days, suggesting population spike potentiation decays faster than field EPSP LTP (Barnes, 1979). The synergistic effects of mTAP-CA1 field EPSP and population spike potentiation may facilitate the mnemonic functions of the mTAP-CA1 activity.

Long-lasting LTP at mTAP-CA1 synapses is significant for several reasons. For instance, mTAP-CA1 LTP may produce stable fields of location specific firing by CA1 place cells in familiar environments (O’Keefe and Nadel, 1978; Thompson and Best, 1990). Previous studies indicate the TAP is crucial for stable place fields (McNaughton et al., 1989; Mizumori et al., 1989; Brun et al., 2002, 2008; Nakashiba et al., 2008). Interestingly, place field stabilization involves mechanisms of synaptic plasticity (Kentros et al., 1998; Rotenberg et al., 2000) and allows a given place field to persist more than 5 months (Thompson and Best, 1990). Thus, plasticity at the TAP-CA1 synapse may underlie the spatial input dependance and environment-specific longevity of stable place cell firing. Future studies are needed to determine if mTAP-CA1 LTP mediates prior observations of preserved incremental spatial learning (Nakashiba et al., 2008) and spatial recognition memory (Brun et al., 2002) in animals with impaired CA3-CA1 neurotransmission.

In summary, this study demonstrates monosynaptic TAP-CA1 responses can be selectively investigated within intact animals. Unlike *in vitro* examinations that offer limited analysis of selective medial or lateral TAP-CA1 LTP induction and longevity, we show selective stimulation of medial TAP afferents to CA1 induces a LTP that is input specific, NMDA receptor-dependent, and persists over 2 weeks in behaving animals. Our observations of mTAP-CA1 responses that appear to contain putative population spikes contrast previous interpretations that TAP inputs are weakly excitatory (Levy et al., 1995), and support previous studies (McNaughton et al., 1989; Mizumori et al., 1989; Brun et al., 2002) suggesting direct TAP-CA1 afferents are capable of discharging CA1 pyramidal cells.

## Author Contributions

JG collected and analyzed data, drafted the manuscript, prepared figures and contributed in manuscript revising and final approval. DMV assisted in data collection, manuscript revising and final approval. ISM assisted in data collection, manuscript revising and final approval. BED contributions include research design, data collection, manuscript revising and final approval.

## Conflict of Interest Statement

The authors declare that the research was conducted in the absence of any commercial or financial relationships that could be construed as a potential conflict of interest.
